# TRIP13/FLNA Complex Promotes Tumor Progression and Is Associated with Unfavorable Outcomes in Melanoma

**DOI:** 10.1155/2022/1419179

**Published:** 2022-10-11

**Authors:** Wang Lu, Zhu Mengxuan, Ren Ming, Gao Zixu, Zhang Yong, Zhang Simin, Yang Yang, Qian Leqi, Shen Kangjie, Li Yanlin, Feng Jia, Ding Yiteng, Wei Chuanyuan, Gu Jianying

**Affiliations:** ^1^Department of Plastic Surgery, Zhongshan Hospital, Fudan University, Shanghai 200032, China; ^2^Department of Medical Oncology, Zhongshan Hospital, Fudan University, Shanghai 200032, China

## Abstract

Cutaneous melanoma is a high-grade malignant tumor originating from skin melanocytes with high risk of recurrence and metastasis. Further study on the mechanism of melanoma development is urgently needed. Here, we performed a bioinformatic analysis to identify critical genes in melanoma using public datasets in the Gene Expression Omnibus database. Among these differentially expressed genes, thyroid hormone receptor interactor 13 (TRIP13) has been reported to exert an important role in the development of various tumors, while its role in melanoma remains unclear. We selected TRIP13 as a candidate gene for further study. TRIP13 expression in clinical specimens was evaluated by immunohistochemistry, and its association with patient prognosis was analyzed by the Kaplan-Meier method and log-rank test. MV3 and A2058 melanoma cells were transfected with lentiviral vector to overexpress or knockdown TRIP13 expression level, and then, its biological function was studied using a series of in vitro and in vivo assays. RNA sequencing, co-immunoprecipitation, and mass spectrometry were used to identify the underlying mechanism of TRIP13. The results of this study exhibited that TRIP13 expression was upregulated in melanoma tissue compared with normal tissues, and high levels of TRIP13 were closely correlated with poor prognoses of melanoma patients. Elevated TRIP13 promoted the invasion and migration of melanoma cells in vitro and enhanced lung metastasis in vivo, without an influence on tumor growth. Importantly, elevated TRIP13 promoted the epithelial-mesenchymal transition (EMT) of melanoma cells, indicating a higher metastatic potential of these cells. Mechanically, TRIP13 physically interacted with filamin A (FLNA) and then activated the PI3K/AKT pathway to transcriptional activation of EMT-related genes. The present study revealed that TRIP13 is a novel prognostic biomarker and potential therapeutic target for melanoma treatment.

## 1. Introduction

Melanoma originates from melanocytes, which is a rapidly growing cancer, and its incidence rates raise approximately 3% yearly [1]. There are about 200,000 new cases and 55,000 deaths from melanoma worldwide each year [2]. Recurrent somatic mutations across all types of melanoma have been identified in genes that regulate proliferation (e.g., BRAF and NRAS) [3], influence growth and metabolism (PTEN and KIT) [4], confer resistance to apoptosis (TP53) [5], and participate in cell cycle regulation (CDKN2A) [6]. Drugs targeting these mutated genes have been successfully developed for the treatment of melanoma, but the prognosis of melanoma patients is still poor due to drug resistance [7, 8]. Therefore, it is very necessary to further study the mechanism of occurrence and development of melanoma in order to explore new targets and new treatment options.

Family members of ATPase associated with diverse cellular activity (AAA ATPase) are widely expressed in multiple tissues and organs and regulate diverse cellular activities including DNA replication, protein degradation, membrane fusion, signal transduction, and gene expression [9]. Recently, AAA ATPase has also been reported to play an important role in tumor development and progression [10, 11]. For example, suppression of ATPase family AAA domain-containing 3A (ATAD3A) by the upregulation of the microRNA miR-210-5P was shown to induce mitochondrial autophagy and sorafenib resistance in hepatoma cells [12]. A recent analysis of melanoma gene expression datasets (GSE3189 and GSE7553) identified differentially expressed genes (DEGs) that were used to establish a protein-protein interaction network in melanoma, and the AAA ATPase family protein thyroid hormone receptor interactor 13 (TRIP13) showed a relatively high degree of connectivity and associated with poor survival [13]. However, the detailed function of TRIP13 in melanoma progression remains unclear.

In view of this, we examined the expression and clinical significance of TRIP13 in tumor tissues of melanoma patients and investigated the biological function and mechanism of TRIP13 in melanoma.

## 2. Materials and Methods

### 2.1. Identification of DEGs and Survival Analysis

We used the following search criteria: “expression profiling by array” (filter), “Homo sapiens” (organism), and “melanoma” (title), and then, three datasets, GSE3189, GSE7553, and GSE15605, met the requirements and were selected for differential gene expression analysis between primary melanoma and normal skin. The GSE3189, GSE7553, and GSE15605 datasets were downloaded from the Gene Expression Omnibus (GEO) database (https://www.ncbi.nlm.nih.gov/geo). Gene expression profiles were analyzed with the Limma package of R software, with a threshold of *p* < 0.05 and log_2_|fold change| > 1.3. GEO2R (https://www.ncbi.nlm.nih.gov/geo/geo2r/) was used to calculate adjusted *p* values and log_2_ fold change (FC) values. Gene Expression Profiling Interactive Analysis (GEPIA; http://gepia.cancer-pku.cn/index.html) using The Cancer Genome Atlas (TCGA) data and Genotype Tissue Expression was used to identify DEGs between cancerous and adjacent normal tissue and for patient survival analysis [14]. TRIP13 expression in melanoma and overall survival (OS) data were obtained from GEPIA.

### 2.2. Patients and Follow-Up

A total of 123 paraffin-embedded melanoma and matched peritumor tissue microarrays (TMAs) were analyzed by immunohistochemistry (IHC). All patients underwent complete excision, and tissue specimens were verified by pathologic examination. The patients did not receive radiotherapy or chemotherapy before the operation. The clinical stage of patients was determined by tumor-node-metastasis staging according to the American Joint Committee on Cancer classification system (8th edition). Ethics approval was obtained from the Ethics Committee of the Zhongshan Hospital Biomedical Research Department, and all patients signed informed consent forms.

### 2.3. IHC

Tissue slides were deparaffinized in xylene and rehydrated in graded alcohol. After antigen retrieval in 1 mM EDTA buffer (pH 8.0), the slides were incubated in 0.3% H_2_O_2_ to block endogenous peroxidase activity. The tissue sections were sequentially incubated with goat serum, an antibody against TRIP13 (sc-514314, 1 : 50) or filamin A (FLNA) (sc-17749, 1 : 50) (both from Santa Cruz Biotechnology, Santa Cruz, CA, USA), and horseradish peroxidase- (HRP-) conjugated anti-mouse IgG (ab205719, 1 : 2000; Abcam, Cambridge, MA, USA) and then stained with diaminobenzidine (Beyotime, Shanghai, China). Immunoreactivity was graded as previously described [15]. Briefly, the score was determined based on a combination of percent staining (1 [0–25%], 2 [>25%–50%], 3 [>50%–75%], or 4 [>75%–100%]) and staining intensity (0 [negative], 1 [low], 2 [moderate], or 3 [high]). The sum of the 2 scores for each specimen was taken as the expression level of TRIP13 or FLNA, which was categorized as low (0–3) or high (4–7).

### 2.4. Cell Culture and Transfection

A2058 and MV3 melanoma cell lines were purchased from the cell bank of the Chinese Academy of Sciences (Shanghai, China) and cultured under the recommended conditions. A2058 cells—which harbor BRAF (V600E), PTEN, and p53 oncogenic mutations—were transfected with pSLenti-EF1a-EGFP-P2A-Puro-CMV-MCS-3 × Flag or pRLenti-EF1a-EGFP-P2A-Puro-CMV-TRIP13-3 × Flag lentiviral vectors (Obio Technology, Shanghai, China). A short hairpin RNA (shRNA) targeting *TRIP13* was synthesized and inserted into the GV493 lentiviral vector (hU6-MCS-CBh-gcGFP-IRES-puro; GeneChem, Shanghai, China). The *TRIP13* shRNA1 sequence was CCCATCGATTTGAGTGCAT, and the *shRNA2* sequence was GCTGAATTCCATGGGCTTT. Transfection efficiency was evaluated by western blotting. A small interfering RNA against FLNA with the sequence AGAAGAAGAUGCACCGCAA was designed and synthesized by Genomeditech (Shanghai, China).

### 2.5. Western Blotting and Immunofluorescence Analysis

Western blotting was performed as previously described [16]. Briefly, melanoma cells were lysed, and 30 *μ*g protein from each sample was separated by 10% sodium dodecyl sulfate polyacrylamide gel electrophoresis and transferred to a polyvinylidene difluoride membrane that was blocked with 5% bovine serum albumin and incubated with primary antibodies against glyceraldehyde 3-phosphate dehydrogenase (GAPDH; #5174, 1 : 1000), protein kinase B (AKT; #4685, 1 : 1000), and phosphorylated (p-)AKT (#4060S, 1 : 1000) (all from Cell Signaling Technology, Danvers, MA, USA); TRIP13 (sc-514314, 1 : 1000), E-cadherin (sc-8426, 1 : 1000), and FLNA (sc-17749, 1 : 1000) (all from Santa Cruz Biotechnology); and N-cadherin (ab18203, 1 *μ*g/ml; Abcam). HRP-conjugated anti-rabbit IgG (#7074, 1 : 1000) and anti-mouse IgG (#7076, 1 : 1000) (both from Cell Signaling Technology) were used as secondary antibodies. Protein bands on the membrane were visualized by enhanced chemiluminescence with a Qinxiang imaging system (Qinxiang, China).

For immunofluorescence analysis, the cells were fixed with 4% formaldehyde for 15 min, permeabilized with 0.3% Triton X-100 (Beyotime) for 30 min at room temperature, and blocked with 5% bovine serum albumin. The cells were incubated overnight at 4°C with primary antibodies, washed 3 times with phosphate-buffered saline (PBS), and incubated with fluorescent secondary antibodies. They were then washed with PBS, stained with DAPI, and observed with a confocal fluorescence microscope (Olympus, Tokyo, Japan).

### 2.6. Transwell and Wound Healing Assays

For the transwell invasion assay, Matrigel (BD Biosciences, Franklin Lakes, NJ, USA) was evenly spread in a transwell chamber and overlaid with 10^4^ cells in 200 *μ*l serum-free Dulbecco's Modified Eagle's Medium (DMEM); medium containing 20% fetal bovine serum was added to the lower chamber. After 48 h, the chamber was removed and washed with PBS, then fixed in 4% paraformaldehyde solution for 30 min, and stained with crystal violet for 15 min. The samples were photographed under a microscope. For the wound healing assay, when cells reached confluence, a 200 *μ*l pipette tip was used to scratch the bottom of the plate; serum-free DMEM was added, and the cells were photographed and then cultured in an incubator at 37°C and 5% CO_2_. The cells were photographed again at 48 h, and the migration rate was calculated using ImageJ software (National Institutes of Health, Bethesda, MD, USA).

### 2.7. In Vivo Assay

For the metastasis model, 10^6^ cells were injected into the tail vein of nude mice (*n* = 6 per group). The mice were euthanized 21 days after injection; the lungs were dissected, fixed with 10% formaldehyde, sectioned, stained with hematoxylin and eosin, and photographed. Lung metastases were counted under the microscope. 10^6^ cells were inoculated subcutaneously on the back of the nude mice, and the nude mice were sacrificed 20 days later and the tumors were weighed.

### 2.8. Co-Immunoprecipitation (co-IP) and Mass Spectrometry (MS)

Melanoma cells were collected in IP lysis solution and centrifuged. Primary antibody and protein A/G beads were added and mixed with the cell lysate at 4°C for 4 h. The precipitate was resuspended in 40 *μ*l loading buffer, and co-IP was performed according to a standard protocol. For liquid chromatography-tandem MS, samples were analyzed using the EASY-nLC 1200 system (Thermo Fisher Scientific, Waltham, MA, USA) and MaxQuant 1.6.1.0 MS software.

### 2.9. Statistical Analysis

Data are expressed as mean ± standard deviation. Student's t test and one-way analysis of variance were used to evaluate the statistical significance of differences between 2 groups and ≥3 groups, respectively. The Kaplan-Meier method and log-rank test were used to analyze OS and disease-free survival (DFS). Independent prognostic factors were analyzed by Cox's proportional hazards regression model. Differences with a *p* value < 0.05 were considered statistically significant.

## 3. Results

### 3.1. TRIP13 Is Highly Expressed and Associated with Poor Prognosis in Melanoma

**Figure 1:**
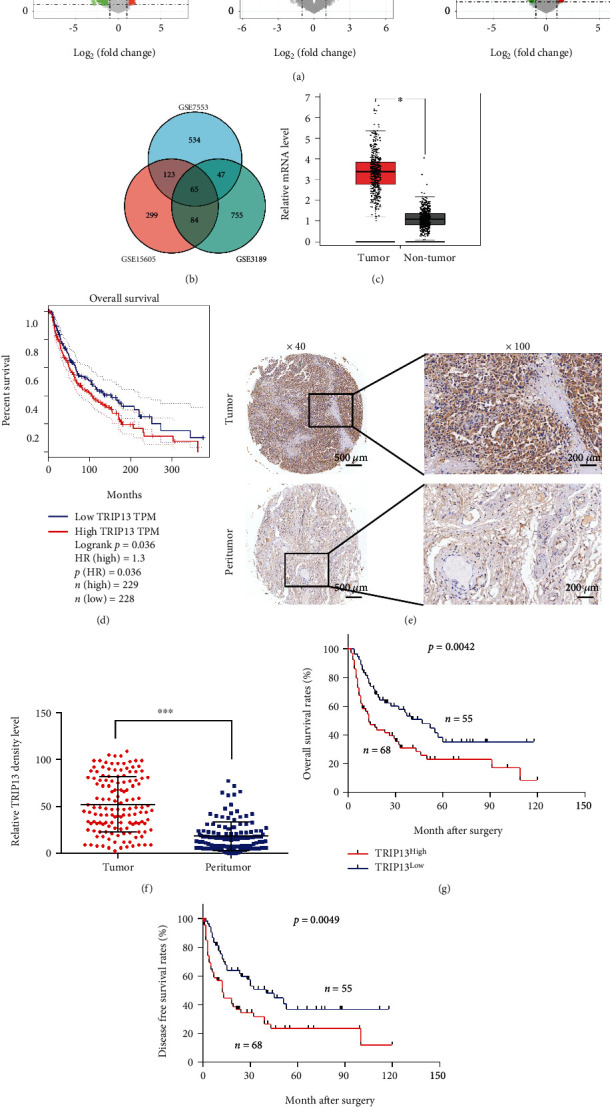
TRIP13 is highly expressed in melanoma, which is associated with poor prognosis. (a) Volcano plot of DEGs in GSE3189, GSE7553, and GSE15605. (b) A total of 65 DEGs (log_2_FC > 1.3, *p* < 0.05) were found to overlap between GSE3189, GSE7553, and GSE15605. (c) *TRIP13* mRNA expression in melanoma and adjacent normal tissue from GEPIA. (d) Survival analysis according to *TRIP13* expression in melanoma patients from GEPIA. (e, f) TRIP13 expression detected by IHC in a TMA containing 138 melanoma patients. (g, h) OS and DFS of 123 melanoma patients according to *TRIP13* expression. ^∗∗∗∗^*p* < 0.0001.

To explore oncogenes that play a key role in melanoma, we screened public databases for melanoma-containing gene sets. GSE3189 contains 45 primary melanoma and 7 normal skin samples; GSE7553 contained 14 primary melanoma and 4 normal skin samples; and GSE15605 contained 46 primary melanoma and 16 normal samples, respectively. After analysis, we obtained 7180 DEGs (2841 upregulated and 4339 downregulated) from GSE3189, 2857 DEGs (1502 upregulated and 1355 downregulated) from GSE7553, and 8173 DEGs (4428 upregulated and 3745 downregulated) from GSE15605 (Figure 1(a)). By taking an intersection of the upregulated DEGs in all the three gene sets, we obtained a total of 65 genes that were commonly upregulated in these 3 datasets (Figure 1(b)). Due to the lack of patient prognostic data in the above datasets, we used the TCGA-SKCM dataset to analyze the prognosis of these patients. Twenty-four of these DEGs were associated with poor prognosis in melanoma patients. One of these genes, TRIP13, which has a role in melanoma remains unclear was selected for further study. In the TCGA-SKCM dataset, we found that *TRIP13* mRNA level was higher in melanoma than in normal skin, and elevated TRIP13 was correlated with poor prognosis of melanoma patients (Figures 1(c) and 1(d)). Then, we examined TRIP13 expression in our own melanoma cohort and found that the protein was localized in the cytoplasm of melanoma cells (Figure 1(e)). TRIP13 was more highly expressed in tumor lesions than in adjacent normal tissues (Figures 1(e) and 1(f)). Importantly, patients with high TRIP13 expression (*n* = 68) had shorter OS (*p* = 0.0042) and DFS (*p* = 0.0049) than those with low expression (*n* = 55) (Figures 1(g) and 1(h)). Additionally, to explore the clinical value of TRIP13, we divided these 123 melanoma patients into two groups according to the expression of TRIP13. We found that high TRIP13 expression was associated with higher malignant phenotype, like more lymph node metastasis (*p* = 0.002) and distant metastasis (*p* = 0.024) (Table 1). Univariate and multivariate analyses showed that high TRIP13 level was an independent predictor of poor prognosis in patients with melanoma (Table 2). Together, these results show that TRIP13 is highly expressed in melanoma and indicated poor prognosis of melanoma patients.

### 3.2. TRIP13 Promotes Melanoma Cell Invasion and Migration In Vitro and In Vivo

**Figure 2:**
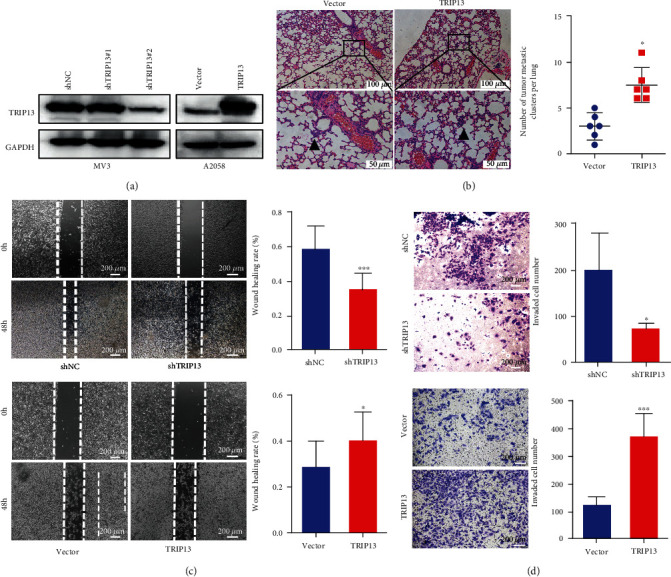
TRIP13 promotes melanoma cell invasion and migration in vitro and in vivo. (a) Transfection efficiency was detected by western blotting. (b) A2058-shNC and A2058-TRIP13 cells were injected into the tail vein of nude mice (*n* = 6). After 21 days, the lungs were dissected and metastatic lesions in the lungs were counted. (c) The migratory ability of melanoma cells with *TRIP13* overexpression or knockdown was evaluated with the wound healing assay. (d) The invasive capacity of melanoma cells with *TRIP13* overexpression or knockdown was assessed with the transwell assays. ^∗^*p* < 0.05, ^∗∗^*p* < 0.01, ^∗∗∗^*p* < 0.001, and ^∗∗∗∗^*p* < 0.0001.

To investigate the biological function of TRIP13 in melanoma cells, we transfected TRIP13 shRNAs into MV3 cells and TRIP13 cDNA vectors into A2058 cells. The transfection efficiency was verified by western blotting (Figure 2(a)). By performing wound healing and transwell migration assays, we found that *TRIP13* knockdown decreased the migration and invasion ability of MV3 cells, while *TRIP13* overexpression increased the migration and invasion ability of A2058 cells (Figures 2(c) and 2(d)). Next, we investigated the role of TRIP13 in tumorigenesis in vivo. By inducing a mouse model of pulmonary metastasis by tail vein injection of A2058 cells, we found that more metastatic lesions were presented in the TRIP13-overexpressing group compared with the control group (Figure 2(b)). However, elevated TRIP13 expression did not influence the tumor growth of melanoma in vivo (Supplementary Figure S1). These results demonstrate that *TRIP13* upregulation mainly promotes melanoma migration and invasion in vivo and in vitro.

### 3.3. TRIP13 Promotes Epithelial-Mesenchymal Transition (EMT) of Melanoma

**Figure 3:**
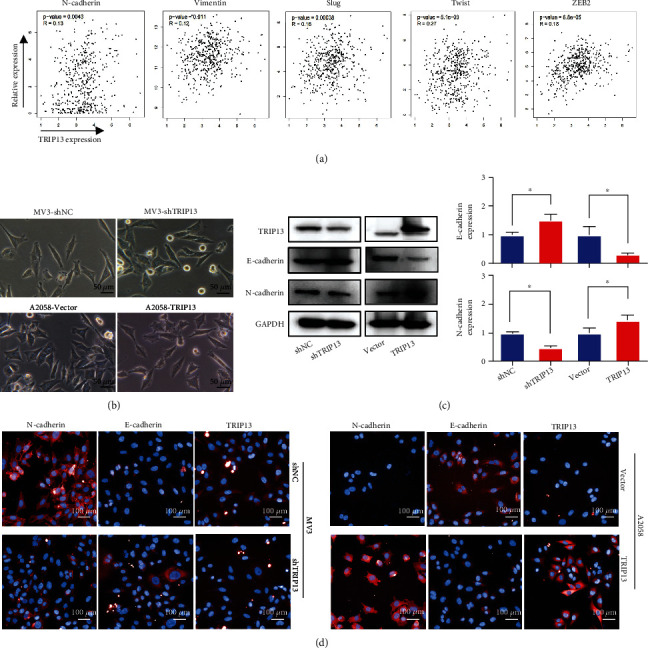
TRIP13 induces EMT in melanoma cells. (a) Relationship between N-cadherin, vimentin, Slug, Twist, zinc finger E-box binding homeobox 2 (ZEB2), and TRIP13 expression in patients with melanoma in TCGA database. (b) Shape of melanoma cells with *TRIP13* overexpression or knockdown. (c, d) Western blot and immunofluorescence analyses of E-cadherin and N-cadherin expressions in A2058 and MV3 cells expressing *TRIP13*-negative control shRNA (A2058-shNC) or empty vector (MV3-Vector), or *TRIP13* overexpression (A2058-TRIP13) or shRNA (MV3-shTRIP13). ^∗^*p* < 0.05.

Since EMT of tumor cells often leads to the enhancement of their migration and invasion ability, we performed correlation analysis using the TCGA database and found that TRIP13 expression was positively correlated with several EMT markers, as N-cadherin (*p* = 0.0043, *r* = 0.13), vimentin (*p* = 0.011, *r* = 0.12), Snail (*p* = 0.00038, *r* = 0.16), Twist (*p* = 5.1e − 09, *r* = 0.27), and ZEB2 (*p* = 6.8e − 05, *r* = 0.18) (Figure 3(a)). Meanwhile, MV3-shTRIP13 and A2058-Vector cells showed epithelial characteristics, whereas MV3-shNC and A2058-TRIP13 cells exhibited features of interstitial cells (Figure 3(b)). Using immunoblot assays, we showed that TRIP13 overexpression increased N-cadherin level and decreased E-cadherin level, whereas *TRIP13* knockdown showed an opposite effect (Figure 3(c)). Furthermore, we performed immunofluorescence analysis and confirmed that TRIP13 promoted the expression of mesenchymal cells and inhibited the expression of epithelial cells (Figure 3(d)). These results show that elevated expression of TRIP13 promotes EMT in melanoma.

### 3.4. Overexpression of TRIP13 Leads to EMT via Activation of the PI3K/AKT Pathway

**Figure 4:**
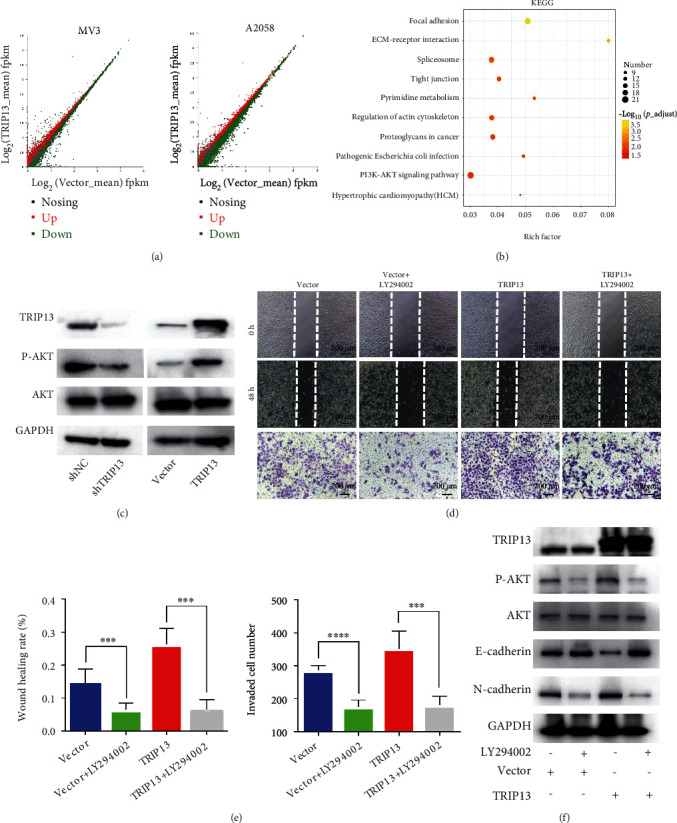
TRIP13 promotes melanoma cell migration, invasion, and EMT via the PI3K/AKT pathway. (a) DEGs in A2058 and MV3 cells expressing *TRIP13*-negative control shRNA (A2058-shNC) or empty vector (MV3-Vector), or *TRIP13* overexpression (A2058-TRIP13) or shRNA (MV3-shTRIP13) depicted by volcano plots. (b) Kyoto Encyclopedia of Genes and Genomes pathway analysis of DEGs. (c) Protein levels of TRIP13, p-AKT, and AKT in A2058-shNC, A2058-TRIP13, MV3-Vector, and MV3-shTRIP13 cells detected by western blotting. (d, e) Migration and invasion of A2058-shNC and A2058-TRIP13 cells treated with LY294002. (f) TRIP13, p-AKT, AKT, E-cadherin, and N-cadherin levels in A2058-shNC and A2058-TRIP13 cells treated with LY294002 detected by western blotting. ^∗∗∗^*p* < 0.001 and ^∗∗∗∗^*p* < 0.0001.

To elucidate the mechanism of TRIP13-induced tumor progression, we performed RNA sequencing on melanoma cells with different TRIP13 expression (Figure 4(a)). There were 399 upregulated and 415 downregulated DEGs in *TRIP13*-depleted MV3 cells and 1481 upregulated and 1640 downregulated DEGs in *TRIP13*-overexpressing A2058 cells compared to the respective control cells. We obtained all the DEGs in TRIP13/Vector-A2058 cells and control/shTRIP13-MV3 cells and took an intersection of these upregulated genes and downregulated genes. There were 452 shared DEGs, and the Kyoto Encyclopedia of Genes and Genomes (KEGG) pathway analysis revealed that they were involved in cancer, focal adhesion, ECM-receptor interaction, regulation of the actin cytoskeleton, and PI3K/AKT signaling (Figure 4(b)). To this, we further speculated that elevated TRIP13 activates the PI3K/AKT signaling. Through immunoblotting assays, we found that high levels of TRIP13 increased the level of p-AKT, whereas decreased TRIP13 had the opposite effect, without an effect on the total AKT level (Figure 4(c)). Additionally, we performed a series of rescue assays and found that the increment in cell migration and invasion induced by TRIP13 overexpression was reversed by treatment with the PI3K/AKT pathway inhibitor LY-294002 (50 *μ*M, 24 h) (Figures 4(d) and 4(e)). Similarly, levels of the EMT markers and p-AKT/AKT were also reversed by the treatment of LY-294002 (Figure 4(f)). Thus, we indicated that TRIP13 induces EMT and tumor progression via the PI3K/AKT signaling.

### 3.5. TRIP13 Binds to FLNA and Activates the PI3K/AKT Signaling

**Figure 5:**
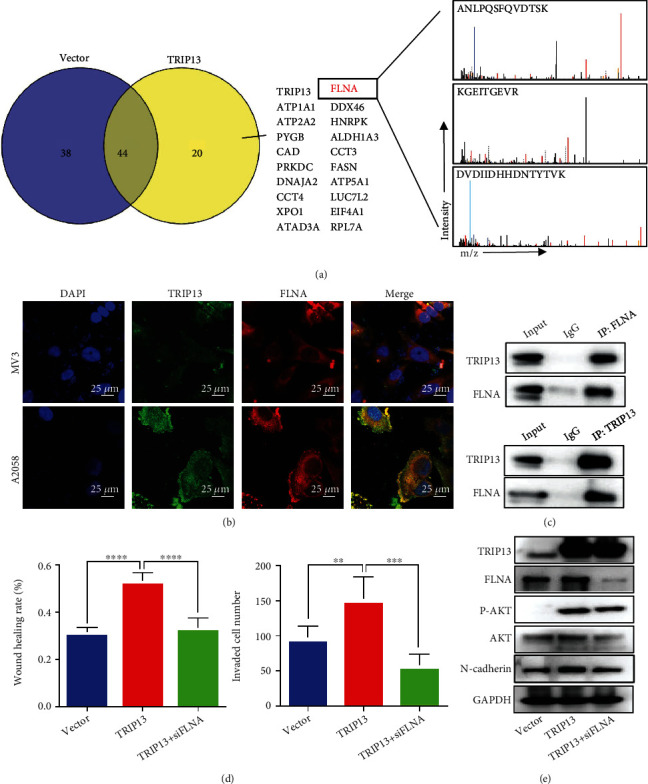
TRIP13 binds to FLNA and activates the PI3K/AKT pathway. (a) Detection of TRIP13-interacting proteins in A2058 cells with *TRIP13* overexpression by co-IP and MS. (b) Colocalization of TRIP13 and FLNA in melanoma cells detected by immunofluorescence labeling. (c) Interaction of TRIP13 and FLNA confirmed by co-IP. (d) Migratory and invasive abilities in transfected cells. (e) TRIP13, FLNA, p-AKT, AKT, and N-cadherin levels in transfected cells as determined by western blotting. ^∗^*p* < 0.05, ^∗∗^*p* < 0.01, ^∗∗∗^*p* < 0.001, and ^∗∗∗∗^*p* < 0.0001.

To further study the molecular mechanism of TRIP13, we performed immunoprecipitation (IP) and liquid chromatography tandem mass spectrometry (LC-MS/MS) to identify the TRIP13-interacting proteins in melanoma. After analyzing the enriched proteins by mass spectrometry, we obtained 82 proteins in A2058-control cells and 64 proteins in TRIP13-overexpressing cells (Figure 5(a)), and only 20 proteins were specifically bound to TRIP13, including FLNA, ATP1A1, and DDX46. One of them, FLNA has been reported to regulate PI3K/AKT pathway and promote EMT of tumor cells [17, 18]. Therefore, we speculated that TRIP13 exerted the role of the PI3K/AKT pathway activation and EMT of melanoma cells through interaction with FLNA. To confirm the interaction between TRIP13 and FLNA, we performed immunofluorescence assays and confirmed that TRIP13 and FLNA colocalized in melanoma cells (Figure 5(b)). co-IP assays further confirmed the physical interaction of TRIP13 and FLNA in melanoma cells (Figure 5(c)). Additionally, to detect the role of FLNA in TRIP13-induced melanoma progression, we performed a series of rescue assays. We showed that FLNA knockdown reversed the increases in cell migration, invasion, EMT, and the PI3K/AKT activation induced by TRIP13 upregulation (Figures 5(d) and 5(e)). These results indicate that TRIP13 activates the PI3K/AKT pathway and induces EMT via interaction with FLNA.

### 3.6. Simultaneous Elevation of TRIP13 and FLNA Predicts Poorer Prognosis in Melanoma Patients

**Figure 6:**
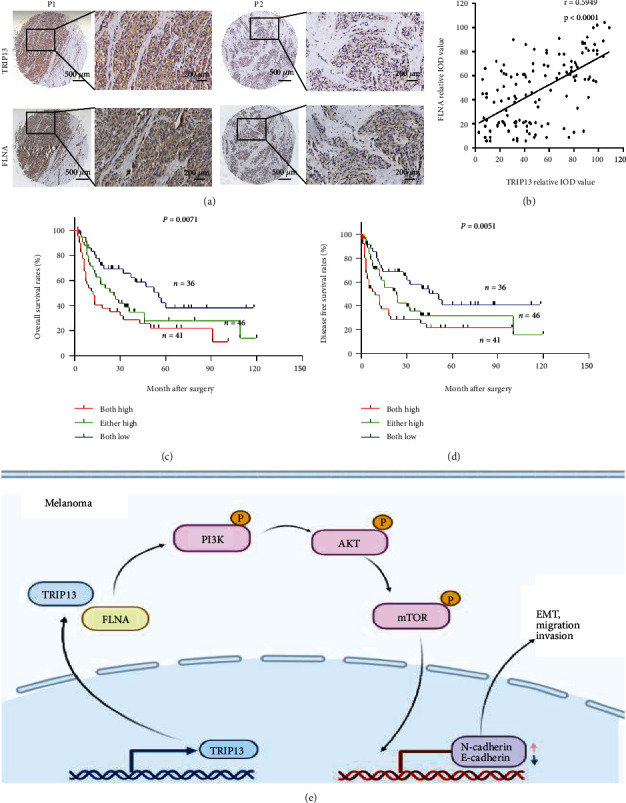
High TRIP13 and FLNA expression is associated with poor prognosis in melanoma patients. (a) TRIP13 and FLNA expression in the TMA detected by IHC. (b) Relationship between FLNA and TRIP13 expression in the TMA. (c, d) OS and DFS analyses of 123 melanoma patients according to TRIP13 and FLNA expression. (e) Model of the biological function and mechanism of TRIP13 in melanoma progression.

We next validated the relationship between TRIP13 and FLNA expression in tissue specimens of melanoma patients. IHC analysis showed a significant positive correlation between TRIP13 and FLNA expression levels (*r* = 0.5949, *p* < 0.0001; Figures 6(a) and 6(b)). Using the Kaplan-Meier analyses, we showed that patients with both high levels of TRIP13 and FLNA had the worst OS (*p* = 0.0071) and DFS (*p* = 0.0051) than those patients with either high or both low levels in melanoma patients (Figures 6(c) and 6(d)), suggesting that the combination of TRIP13 and FLNA expression can serve as an efficient biomarker of unfavorable prognosis in melanoma. Together, we propose a model in which TRIP13 activates the PI3K/AKT signaling to transcriptional activation of N-cadherin and inhibition of E-cadherin through the interaction with FLNA and then induces melanoma cell migration, invasion, and EMT (Figure 6(e)).

## 4. Discussion

In this study, we investigated the biological function and the underlying mechanisms of TRIP13 in melanoma. We found that TRIP13 was significantly upregulated in melanoma compared to adjacent normal tissue, and it was strongly correlated with poor prognosis in melanoma patients. TRIP13 overexpression was associated with a malignant phenotype of melanoma, and the interaction of TRIP13 and FLNA activated the PI3K/AKT pathway to induce EMT in melanoma cells.

TRIP13 plays a key role in regulating mitosis including the spindle assembly checkpoint and DNA repair [19, 20]. Aberrant TRIP13 expression has been observed in various human cancers and was shown to be related to a malignant phenotype—e.g., excessive cell proliferation, drug resistance, and tumor progression [21]. One study found that TRIP13 promoted the proliferation, migration, and invasion and EMT of prostate cancer cells through regulation of tyrosine 3-monooxygenase/tryptophan 5-monooxygenase activation protein zeta (YWHAZ) [22]. TRIP13 also promoted lung adenocarcinoma cell proliferation, clonogenicity, and migration while inhibiting apoptosis and G2/M phase transition in vitro [23]. In line with these studies, we were the first to find that increased TRIP13 expression enhanced melanoma cell invasion and migration and induced EMT via activation of the PI3K/AKT pathway. Additionally, a high TRIP13 level was associated with lymph node metastasis and distant metastasis. Importantly, univariate and multivariate analyses showed that elevated TRIP13 expression was an independent predictor of unfavorable melanoma prognosis. However, in the subcutaneous tumor formation experiment in vivo, we found that TRIP13 had no obvious effect on tumor growth. We speculated that this might be related to the immune microenvironment, local hypoxia of the tumor, and other conditions. One research demonstrated that PTP1B promotes cell migration and invasion in melanoma as an oncogene, while knockdown or overexpression of PTP1B had no effect on the growth of melanoma cells [24]. Our study is similar to theirs. In our study, we found that elevated TRIP13 mainly promoted the invasion and migration ability of melanoma cells using series of in vivo and in vitro assays but had no effect on tumor growth through in vivo experiment. This illustrates that TRIP13 exerts a tumor-promoting role in a variety of tumors, but there may be subtle differences in different tumors.

AAA ATPase facilitates the assembly or degradation of protein complexes [25]. Driven by ATP binding and hydrolysis cycles, conformational changes in AAA+ ATPases can generate mechanical work to unwind substrate proteins for degradation within the central axial channel of the ATPase loop. Several studies showed that TRIP13 as an AAA ATPase, it does not exercise the function of protein degradation but affect the progression of tumors by interacting with some proteins to affect downstream signaling pathway. In hepatocellular carcinoma, TRIP13 regulated by miR-192-5p was shown to interact with and increase the expression of actinin alpha 4 (ACTN4), thus activating the AKT/mammalian target of rapamycin (mTOR) pathway to drive tumor progression [26]. In colorectal carcinoma (CRC), TRIP13 interacted with the receptor tyrosine kinase fibroblast growth factor receptor 4 (FGFR4) and activated the epidermal growth factor receptor (EGFR)/AKT pathway to induce EMT [27]. In the present study, we found that TRIP13 also activates AKT signaling to promote melanoma malignant phenotypes which is consistent with the previous research; we further discovered that TRIP13 activates AKT signaling by directly interacting with FLNA. FLNA, the most abundant and widely expressed member of the filamin family, functions as a nonmuscle actin filament crosslinking protein [28] to stabilize the cytoskeleton and maintain the structural integrity of cells [29]. FLNA was shown to associate with multiple functional noncytoskeletal proteins and is involved in several pathways that regulate cell migration and adhesion and promote tumor progression [30]. For example, c-Met/AKT/FLNA/SMAD formed a positive feedback loop to enhance EMT, which contributed to 5-fluorouracil resistance in CRC cells [31]. Consistent with these observations, we showed that *FLNA* knockdown partly abolished TRIP13-induced EMT and activated PI3K/AKT signaling. Moreover, the Kaplan-Meier analyses showing patients with higher expression of TRIP13 and FLNA had reduced OS and DFS than other patients; it indicates that TRIP13 combined with FLNA can be an effective indicator to predict prognosis in melanoma patients.

## 5. Conclusion

In conclusion, the results of this study demonstrate the important role of TRIP13 in promoting melanoma progression and EMT via the FLNA/PI3K/AKT pathway. These findings provide new insight into the molecular basis of melanoma progression and suggest that TRIP13 is a useful prognostic indicator and potential therapeutic target for melanoma treatment.

## Figures and Tables

**Table 1:** Correlations between TRIP13 with clinicopathologic features in 123 melanoma patients.

Variable	Number of patients		*p* value^∗^
	TRIP13^low^	TRIP13^high^	
Age (year)			
<60	25	24	0.397
≥60	32	42	
Gender			
Male	33	30	0.169
Female	24	36	
Ulceration			
Present	45	58	0.181
Absent	12	8	
Breslow depth (mm)			
≤2	33	29	0.123
>2	24	37	
Clark level			
I-III	33	33	0.381
IV-V	24	33	
Lymph node metastasis			
No	50	42	0.002
Yes	7	24	
Distant metastasis			
No	46	41	0.024
Yes	11	25	
Clinical stage			
I-II	33	42	0.515
III-IV	24	24	

Note: a chi-square test was used for comparing groups between low and high TRIP13 expression.

**Table 2:** Univariate and multivariate analyses of factors associated with overall survival.

Variable	Overall survival			
	Multivariate analysis			
	Univariate *p*	HR	95% CI	*p* ^∗^
Age (year) (≥60 vs. <60)	0.166			NA
Gender (men vs. women)	0.195			NA
Ulceration (present vs. absent)	0.149			NA
Breslow depth (mm) (≤2 vs. >2)	0.543			NA
Clark level (I-III vs. IV-V)	0.104			NA
Lymph node metastasis (yes vs. no)	0.143			NA
Distant metastasis (yes vs. no)	0.003	1.44	0.837-2.478	0.188
Clinical stage (yes vs. no)	0.03	1.59	0.947-2.671	0.079
TRIP13 staining (low vs. high)	0.003	1.933	1.171-3.188	0.01

Note: NA: not adopt; *p* value was calculated using Cox proportional hazards regression.

## Data Availability

The sequencing data are available which can be obtained by request to the corresponding authors.

## Abbreviations

AAA ATPase: ATPase associated with diverse cellular activity
AKT: Protein kinase B
co-IP: Co-immunoprecipitation
CRC: Colorectal carcinoma
DEG: Differentially expressed gene
DFS: Disease-free survival
DMEM: Dulbecco's Modified Eagle's Medium
EMT: Epithelial-mesenchymal transition
FC: Fold change
FLNA: Filamin A
GEO: Gene Expression Omnibus
GEPIA: Gene expression profiling interactive analysis
HRP: Horseradish peroxidase
IHC: Immunohistochemistry
OS: Overall survival
MS: Mass spectrometry
PBS: Phosphate-buffered saline
PI3K: Phosphoinositide 3-kinase
shRNA: Short hairpin RNA
TCGA: The Cancer Genome Atlas
TMA: Tissue microarray
TRIP13: Thyroid hormone receptor interactor 13.

## Supplementary Materials

Supplementary Materials Figure 1: overexpression of TRIP 13 does not influence the tumor growth in vivo. A. 10^6^ cells (A2058-Vector/A2058-TRIP13) were separately inoculated subcutaneously on the back of the nude mice (*n* = 6), and the nude mice were sacrificed 20 days later and the tumors were weighed.
