# Supplementary figures and images for: TRIP13/FLNA Complex Promotes Tumor Progression and Is Associated with Unfavorable Outcomes in Melanoma
Source: J Oncol. 2022 Oct 11;2022:1419179. doi: 10.1155/2022/1419179 (PMC9578791; doi:10.1155/2022/1419179)

**A**

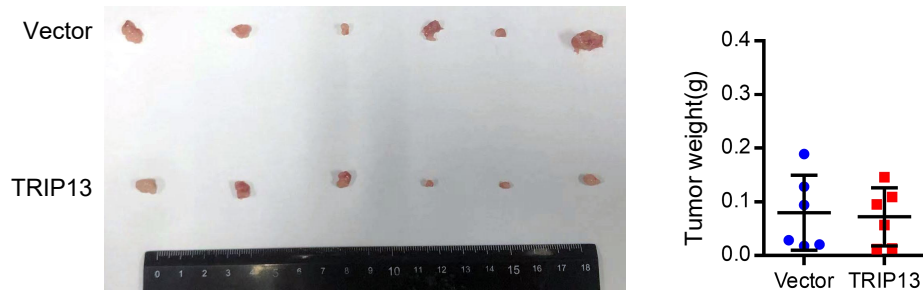

Figure 1. Overexpression of TRIP 13 doesn't influence the tumor growth in vivo. A

Supplement: Supplementary Materials — Figure 1: overexpression of TRIP 13 does not influence the tumor growth in vivo. A. 106 cells (A2058-Vector/A2058-TRIP13) were separately inoculated subcutaneously on the back of the nude mice (n = 6), and the nude mice were sacrificed 20 days later and the tumors were weighed. [file 1419179.f1.pdf]
